# The evolutionary history of manatees told by their mitogenomes

**DOI:** 10.1038/s41598-021-82390-2

**Published:** 2021-02-11

**Authors:** Érica Martinha Silva de Souza, Lucas Freitas, Elisa Karen da Silva Ramos, Giovanna Selleghin-Veiga, Michelle Carneiro Rachid-Ribeiro, Felipe André Silva, Miriam Marmontel, Fabrício Rodrigues dos Santos, Anne Laudisoit, Erik Verheyen, Daryl P. Domning, Mariana Freitas Nery

**Affiliations:** 1 Departamento de Genética, Evolução, Microbiologia e Imunologia, Instituto de Biologia, Universidade Estadual de Campinas, Campinas, Brazil; 2 Instituto de Desenvolvimento Sustentável Mamirauá, Tefé, Brazil; 3 Departamento de Biologia Geral, Universidade Federal de Minas Gerais, Belo Horizonte, Brazil; 4 EcoHealth Alliance, New York, USA; 5 Royal Belgian Institute of Natural Sciences, Brussels, Belgium; 6 Biology Department, Evolutionary Ecology Group, University of Antwerp, Antwerp, Belgium; 7 Laboratory of Evolutionary Biology, Department of Anatomy, Howard University, Washington, D.C. 20059 USA; 8 Research Associate, Department of Paleobiology, National Museum of Natural History, Smithsonian Institution, Washington, D.C. 20560 USA

**Keywords:** Evolution, Evolutionary genetics, Genetics, Evolutionary biology

## Abstract

The manatee family encompasses three extant congeneric species: *Trichechus senegalensis* (African manatee), *T. inunguis* (Amazonian manatee), and *T. manatus* (West Indian manatee). The fossil record for manatees is scant, and few phylogenetic studies have focused on their evolutionary history. We use full mitogenomes of all extant manatee species to infer the divergence dates and biogeographical histories of these species and the effect of natural selection on their mitogenomes. The complete mitochondrial genomes of *T. inunguis* (16,851 bp), *T. senegalensis* (16,882 bp), and *T. manatus* (16,882 bp), comprise 13 protein-coding genes, 2 ribosomal RNA genes (rRNA - 12S and 16S), and 22 transfer RNA genes (tRNA), and (D-loop/CR). Our analyses show that the first split within *Trichechus* occurred during the Late Miocene (posterior mean 6.56 Ma and 95% HPD 3.81–10.66 Ma), followed by a diversification event in the Plio-Pleistocene (posterior mean 1.34 Ma, 95% HPD 0.1–4.23) in the clade composed by *T. inunguis* and *T. manatus*; *T. senegalensis* is the sister group of this clade with higher support values (*pp* > 0.90). The branch-site test identified positive selection on *T. inunguis* in the 181st position of the *ND4* amino acid gene (LRT = 6.06, *p* = 0.0069, BEB posterior probability = 0.96). The *ND4* gene encodes one subunit of the NADH dehydrogenase complex, part of the oxidative phosphorylation machinery. In conclusion, our results provide novel insight into the evolutionary history of the Trichechidae during the Late Miocene, which was influenced by geological events, such as Amazon Basin formation.

## Introduction

**Figure 1:**
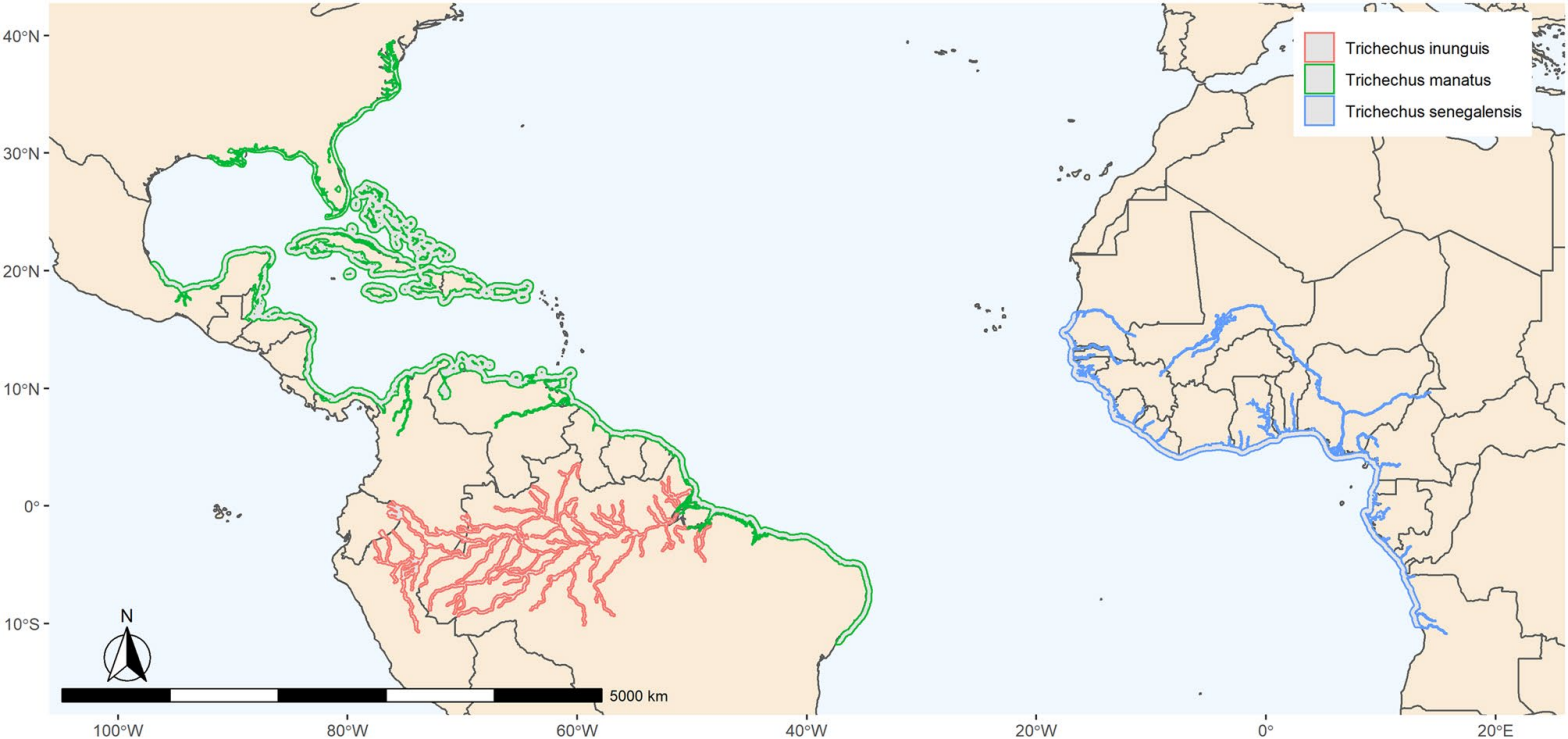
Distribution of *Trichechus* species in the world, map made by EMSS with Rstudio v.1.3 using distribution shapefile from IUCN database, script available at author’s Github webpage (https://github.com/souzaems/scripts/blob/master/map_on_R.r).

The Order Sirenia comprises a group of Afrotherian aquatic mammals that arose by the Early Paleocene^1^ and are currently distributed throughout the Indo-Pacific region and from the southeastern part of the United States of America to part of the Brazilian coast, Amazon region, and African coast^2,3^. Sirenians are classified into two extant families: Trichechidae and Dugongidae. The Family Dugongidae has only one living species (*Dugong dugon* MÜLLER, 1776), because the Steller’s sea cow (*Hydrodamalis gigas* ZIMMERMANN, 1780) was driven to extinction in the eighteenth century due to overhunting^4^. Dugongidae is considered the older of these two families and it was cosmopolitan until its diversity decreased during the Pliocene^5,6^. The modern dugong is restricted to the Indian Ocean and West Pacific region, specifically in regions of high concentration of seagrass^7^. The Family Trichechidae is composed of two subfamilies, Miosireninae (extinct species), and Trichechinae, which includes all modern species^8^. The Subfamily Trichechinae has only one extant genus (*Trichechus*) and three species: *Trichechus senegalensis* LINK, 1795 (African manatee) living along the coasts and rivers of western Africa, *T. inunguis* NATTERER, 1883 (Amazonian manatee) which is endemic to the Amazon Basin, and *T. manatus* LINNAEUS, 1758 (West Indian manatee) ranging from the southeastern USA and the Caribbean region to the Brazilian northeastern coast^9^ (Fig. 1).

The fossil record is scant for the Family Trichechidae, which makes it difficult to infer their evolutionary history^4^. The currently accepted evolutionary scenario for the genus *Trichechus* is that it originated in South American rivers from where it colonized the marine environment and reached the African continent^9–11^. However, when and how the *Trichechus* species diverged are still mainly inferred from fossil age and morphological characteristics. Until recently, few genetic studies have attempted to shed light on their evolution, but these studies used single genetic markers or did not include all representatives of the genus^9,12,13^. One of these studies used a single mitochondrial gene to infer the divergence time between *T. manatus* and *T. inunguis*, which was estimated to have been 2–4 million years ago (Ma), in the Plio-Pleistocene^3,9^.

The mitochondrial genome encodes 13 proteins, which belong to a complex of oxidative phosphorylation pathways (OXPHOS) and have been extensively used to unravel phylogenetic relationships^14–16^. For a long time, the mitochondrial genome was considered to be under neutral or nearly neutral selection^17^. However, growing evidence has revealed that these genes may be subject to positive directional selection^16,18,19^. Hence, more recently, attention has been given to the study of molecular adaptation of mitochondrial genes, and many studies have shown that amino acid substitutions on these proteins may improve aerobic capacity and may be related to adaptation to new environments^20,21^. The aquatic mammals, such as manatees, are textbook examples of lineages that have undergone extreme adaptations related to the transition from land to aquatic environments^3,22^. Accordingly, positive selection in genes such as cytochrome b^23^ and *ND2*, *ND4*, and *ND5*^24^ was already detected in killer whales (*Orcinus orca*, Linnaeus 1758) and dugongs, respectively, but such mitochondrial molecular evolution in manatees has been little studied. In this context, here we sequenced the complete mitogenomes of all extant species of manatees to infer their phylogenetic relationships, to estimate divergence times among the species and to evaluate the effect of natural selection on mitochondrial genes during their evolution.

## Material and methods

### Sampling and DNA extraction

We extracted DNA from one tissue sample of *T. senegalensis* (Democratic Republic of the Congo^12^), one tissue sample of *Trichechus inunguis* (female, Japurá River, Brazil) and one tissue sample from *Trichechus manatus* (male, Ceará, Brazil). All tissues were collected following the respective environmental regulations. The DNA extraction of *T. senegalensis* was done following the Phenol:Chloroform:Isoamyl Alcohol 25:24:1 according to manufacturers. The collecting permits were provided by Instituto Chico Mendes de Conservação da Biodiversidade (ICMBio, number 44628-2), and the re-importation tissue for *T. senegalensis* was provided by CITES number 19BR031212/DF. The activity of access to the Genetic Heritage is registered under the Sistema Nacional De Gestão Do Patrimônio Genético E Do Conhecimento Tradicional Associado (SisGen) number A94D205. This study was carried out according to the Brazilian guidelines for animal care and authorized by the Committee on the Ethics of Animal Use and Care – CEUA of the State University of Campinas – UNICAMP.

The DNA quality and integrity were evaluated on 1% agarose gel. The concentration and purity of samples were verified using the NanoDrop 2000 Spectrophotometer (Thermo Scientific) and subsequently confirmed by fluorimetry on Qubit 2.0 (Invitrogen, Life Technologies). The mitogenome of *T. senegalensis* was sequenced by an external company (BPI Biotecnologia Pesquisa e Inovação) using the MiSeq Illumina Platform.

### mtDNA assembly and annotation

The mitochondrial genomes (mtDNA) from *T. inunguis* and *T. manatus* were retrieved from the whole genome sequenced by the HiSeq 2500 platform. We first created two custom BLAST databases on these genomes. Then we used the mtDNA from *T. manatus* (GenBank access numbers AM904728, and MN105083), as query in BLAST + v. 2.9.0 to search *T*. *inunguis* and *T*. *manatus* mtDNAs^13,25^. We subsequently used a custom Python 3 script^26^ to retrieve BLAST hits and did the assembly with Geneious R9 (https://www.geneious.com). The mtDNA of *T. senegalensis* was assembled using MitoFinder^27^.

We used GeSeq—Annotation of Organellar Genomes server^28^ to annotate mtDNA, and to avoid common annotation errors^29^, we used reference data (*Homo sapiens* NC_012920.1, and *Mus musculus* NC_005089.1) to compare with our annotation. We used OGDRAW—Draw Organelle Genome Maps^28^ for visualization. Finally, we used ARWEN v1.2.3, ARAGORN v1.2.38 and tRNAscan-SE v2.0.3 as implemented in the MITOS web server^30^ and Geseq server^28^ to annotate the tRNA.

### Phylogenetic analyses

To infer trichechid phylogeny, we used the following outgroups: *Dugong dugon* (accession numbers AY075116, and NC003314) as sister group of Trichechidae, *Loxodonta africana* (NC000934), *Loxodonta cyclotis* (JN673263), *Elephantulus edwardii* (NC041486), *Echinops telfairi* (AB099484), and *Dasypus novemcinctus* (Y11832). First, we aligned the coding genes with the MAFFT^31^ algorithm and translated into proteins using Geneious R9 (https://www.geneious.com). The best evolutionary model to be used in phylogenetic analysis was determined by PartitionFinder 2.0^32^ using the Akaike information criterion (AIC); first we tested using all the mitogenome data (tRNAs, rRNA, PCGs), and then only 13 coding genes.

We built a maximum likelihood (ML) tree using RAxML v.8.0, and the GTR + GAMMA model^33^. Also, the Bayesian inference (BI) tree was inferred by MrBayes v.3.2.6^34^ using the models inferred with PartitionFinder. The Bayesian analysis was conducted using the Markov chain Monte Carlo (MCMC) method with three heated and one cold chain, sampling every 1,000,000 generations from 20,000,000 generations, and discarding the first 500,000 generations as burn-in.

We estimated divergence time in BEAST2^35^, with custom parameters for the calibration nodes that were chosen using fossil data (Table 1). The analyses were performed using the following parameters: for the sites—HKY substitution model, with empirical base frequencies, and gamma site heterogeneity model; for tree—Yule Process Speciation model as the tree prior, and a random starting tree; for the clock—a lognormal relaxed clock uncorrelated prior. After setting the parameters, we performed two independent runs with 100,000,000 generations, sampling every 5000 generations. We used Tracer v.1.6.0^36^ to check for convergence of the chains to stationary distributions, then we summarized the runs using LogCombiner v1.8.2^37^. We built the final tree using the combined results from all trees using TreeAnnotator v1.8.2^38^, and we visualized the tree with FigTree v.1.4.2^39^.

**Table 1:** Parameters used to calibrate the divergence time analysis, using fossil data on BEAST2.

Node placement	Specimen	Age (Ma)	Lognormal distribution parameters	References
Afrotheria (A)	*Ocepeia*	59.2–61.6	Offset = 9.0, Mean = 61.6, SD = 0.25	^67^
Macroscelidae-Afrosoricida (B)	*Chambius*	40.4–55.8	Offset = 2.0, Mean = 55.8, SD = 0.25	^68^
Paenungulata (C)	*Phosphatherium*	48.6–55.8	Offset = 2.0, Mean = 55.8, SD = 0.25	^69^
Sirenia (D)	*Prorastomus sirenoides*	46.3–47.3	Offset = 3.0, Mean = 47.3, SD = 0.15	^70,71^
Proboscidea (F)	*Eritreum melakeghebrekristosi*	26.8–27.0	Offset = 4.5, Mean = 27.0, SD = 0.25	^72^
*Trichechus* (G)	*Trichechus* sp.	5.3–11.60	Offset = 3.0, Mean = 11.6, SD = 0.7	^73^

### Adaptive molecular evolution analysis

To identify codon sites with positive selection in the Family Trichechidae, we estimated the ω (*dN*/*dS*) rate, where *dN* is the non-synonymous substitution rate (the rate at which changes in nucleotide sites lead to changes into new amino acids) and *dS* is the synonymous substitution rate (the rate at which changes in nucleotide sites do not lead to changes in the amino acid chain). A ω > 1 indicates positive selection, i.e. when natural selection is favoring amino acid changes. We used the branch-site test for positive selection^40^ implemented in Godon software^41^, which incorporate codon rate variation approaches, and gamma variation between codons^41–43^. We applied this test on the thirteen coding genes of *Trichechus* species and the outgroups. We ran five tests for each gene, labelling the following lineages: (I) *T. manatus* only, (II) *T. inunguis* only, (III) *T. senegalensis* only, (IV) the lineage of *T. manatus* and *T. inunguis* and (V) the entire Genus *Trichechus* (Supplementary Figure 1).

To estimate whether substitutions with signs of positive selection will affect the structure and function of mitochondrial proteins, we aligned the *Trichechus* sp. and got the X-ray crystal structures using SWISS-MODEL^44^. We also estimated domain position using the InterPro web tool^45^.

## Results

### Assembly and annotation

**Figure 2:**
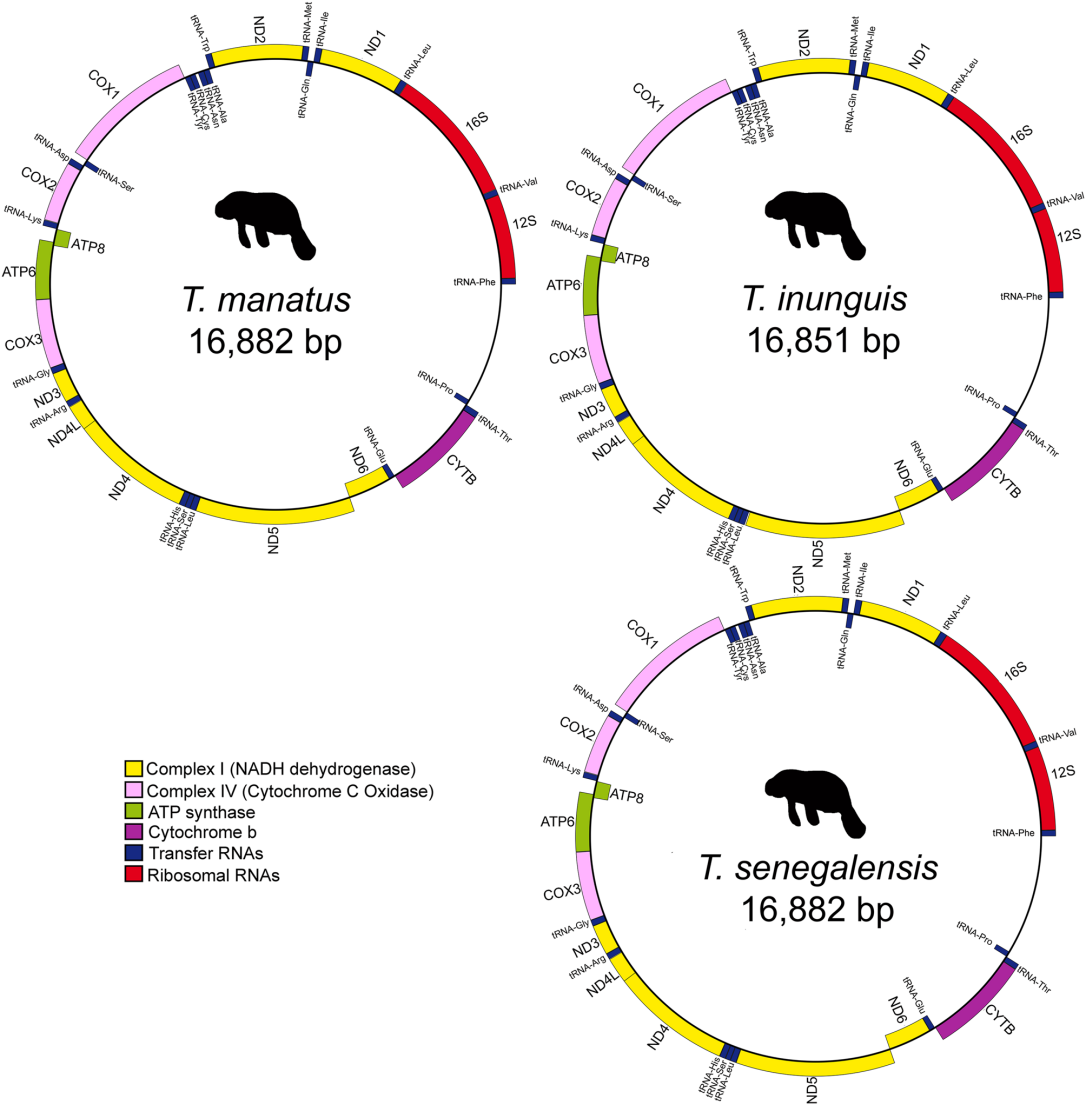
Scheme of circular mitochondrial genome of *Trichechus* species: 13 protein-coding genes, 2 ribosomal RNA genes, 22 transfer RNA genes, and the putative control region.

Here we present the complete mitochondrial genomes of the three contemporaneous Trichechinae species: *Trichechus inunguis* 16,851 base pairs (bp), *T. senegalensis* 16,882 bp, and *T. manatus* 16,924 bp, deposited under GenBank accession numbers MW073826, MW073827 and MW073828, respectively. The mitochondrial genome structure of all *Trichechus* species consists of 13 protein-coding genes, 2 ribosomal RNA genes (rRNA - 12S and 16S), and 22 transfer RNA genes (tRNA). Most of these elements were encoded on the H-strand, except for two protein-coding genes (*ATP8 and ND6*), and eight tRNA (Gln, Ala, Asn, Cys, Tyr, Ser, Glu, Pro) that were encoded in the L-strand; for all species the tRNA length ranges between 59 and 75 bp (Fig. 2, and Supplementary Table 1).

### Phylogenetic analyses

We generated maximum likelihood and Bayesian trees using two types of dataset: one using all mitogenome data, and the second using only the 13 protein-coding genes from the mitogenome. All phylogenetic trees resulted in the same topology, with the highest posterior probability and bootstrap values (Supplementary Figure 2). Our mitochondrial phylogeny depicts *Dugong dugon* as a sister group of all *Trichechus* species, with high posterior probability (*pp*) > 0.99, and bootstrap value of 100. Within the Trichechidae, *T. senegalensis* is the sister group of a clade composed by *T. manatus* and *T. inunguis*, both relationships with strong statistical support in Bayesian analysis (*pp* > 0.95), and bootstrap of 65 in maximum likelihood analysis.

**Figure 3:**
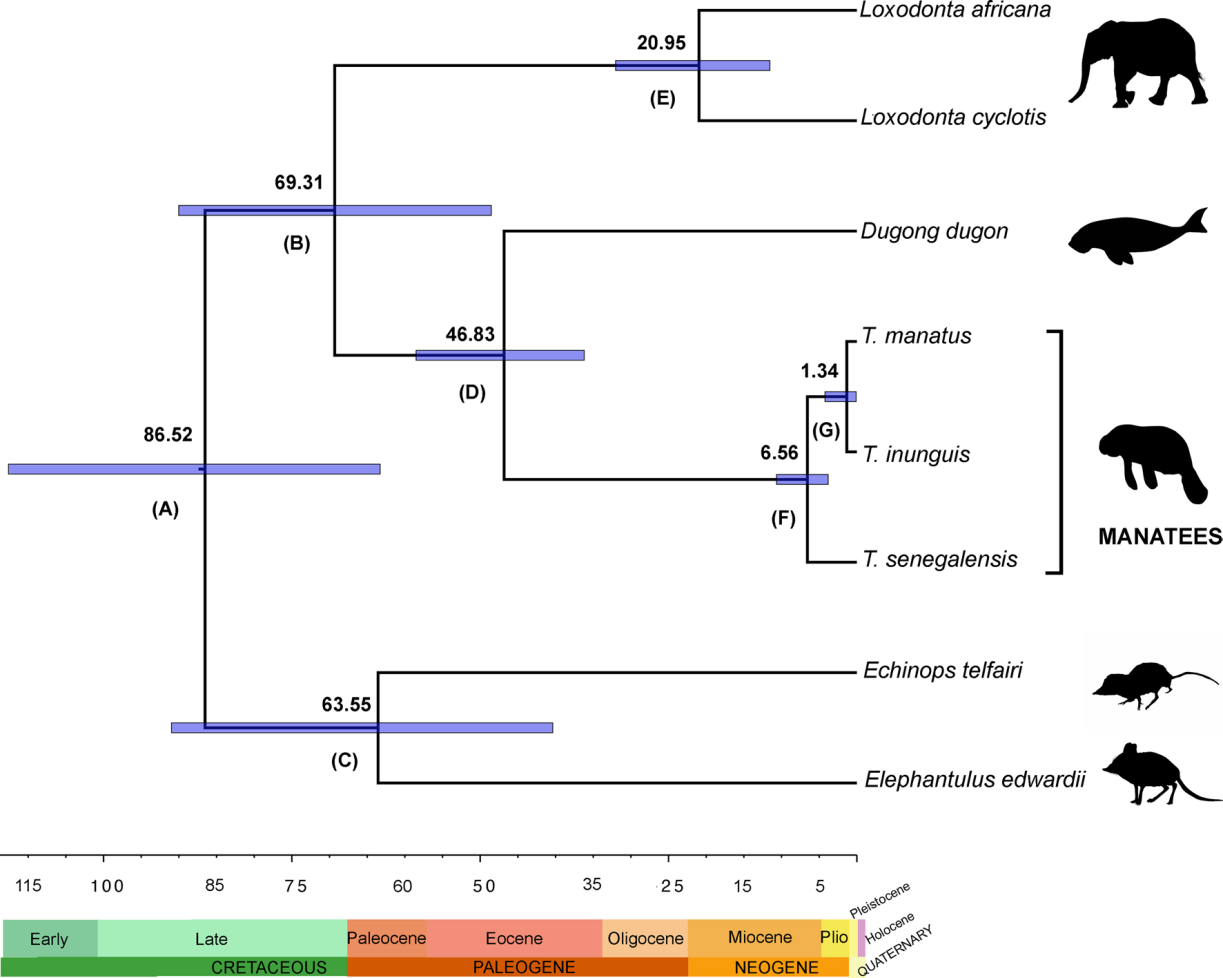
A phylogenetic tree for time divergence between the *Trichechus* species. The purple bars represent the mean age of lineages split.

We estimated the ages of the divergence events within the Trichechidae using nodes of calibration based on the fossil record (Table 2). Our results suggest that the genus *Trichechus* originated during the Late Miocene, 6.56 Ma (95% HPD 3.81–10.66), and the divergence of *T. manatus* and *T. inunguis* may have occurred in the Pleistocene, 1.34 Ma (95% HPD 0.1–4.23) (Fig. 3).

**Table 2:** The values for lineages divergence, the mean ages and 95% highest posterior density range (HPD).

Lineage	Mean age (Ma)	HPD (Ma)
Afrotheria (A)	86.52	63.28–112.62
Macroscelididae-Afrosoricida (B)	63.55	40.37–90.98
Paenungulata (C)	69.31	48.53–90.02
Sirenia (D)	46.83	36.19–58.51
Proboscidea (E)	20.95	11.55–32.02
*Trichechus* (F)	6.56	3.81–10.66
*T. manatus* + *T. inunguis* (G)	1.34	0.1–4.23

### Adaptive molecular evolution

We used the branch-site test to identify positive selection in *Trichechus* species. We detected evidence for positive selection only in the *T. inunguis* lineage, at the 181st position of the *ND4* amino acid gene (LRT = 6.06, *p* = 0.0069, Bayes Empirical Bayes posterior probability = 0.96).

### Protein structure

**Figure 4:**
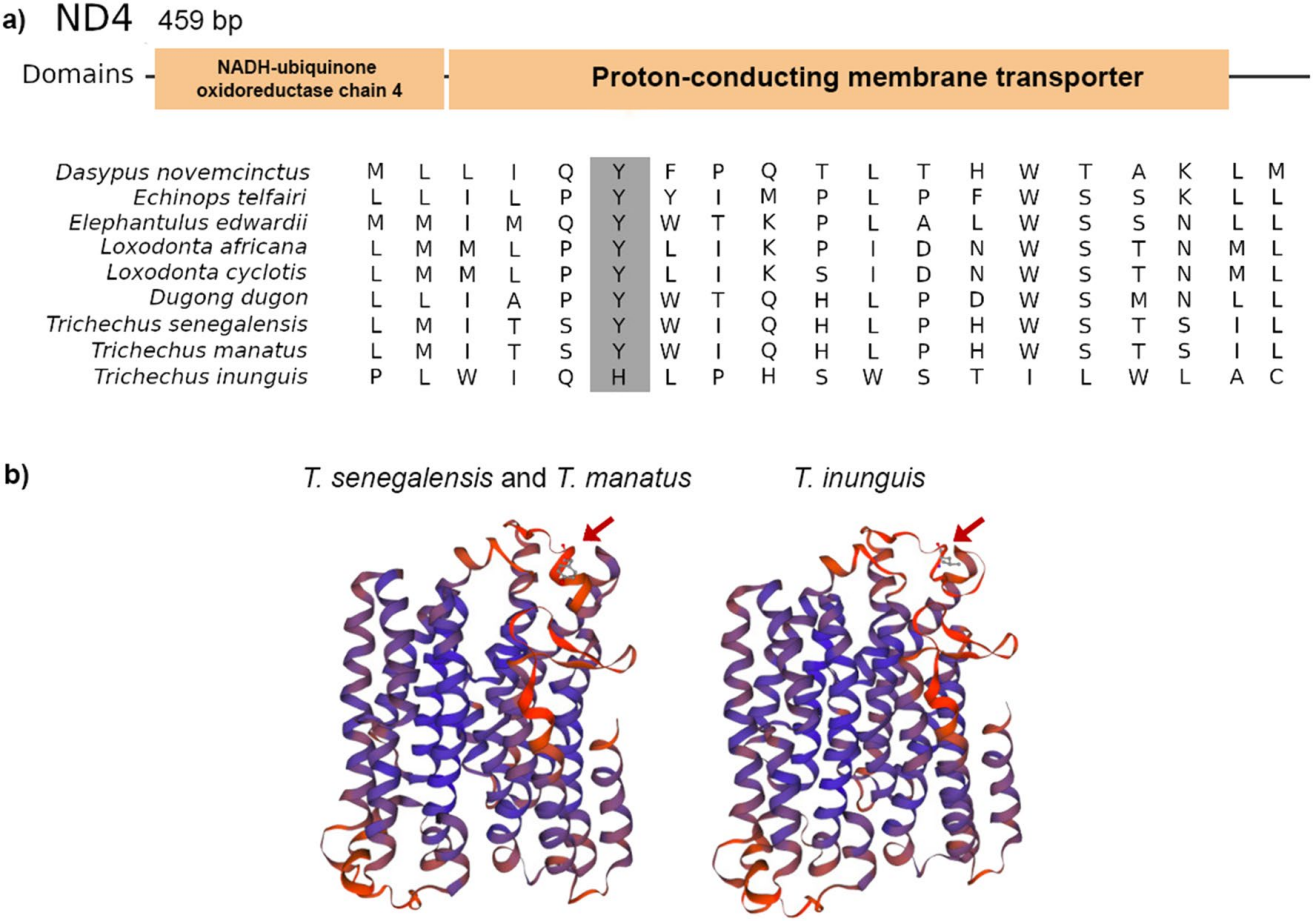
The alignment of *ND4* gene, (**a**) represents the *ND4* domain and the highlighted indicates the amino acid position of positive selection. (**b**) Modelling protein of *ND4* of *Trichechu*s species; the arrow shows the positive selection area.

We mapped the positively selected site identified by branch-site test onto the mitochondrial protein three-dimensional (3D) crystal structures and searched for the proximity of this site to the functional domains of the protein. The alignment of homologous structures for *ND4* and crystal structures revealed that the site with a significant signal of positive selection (site 181) is inside a highly conserved core in the main domain of the *ND4* protein. This region corresponds to the discontinuous region of the transmembrane helices 7 (Fig. 4), which enables flexibility for the *ND4* subunit.

## Discussion

### Mitochondrial DNA annotation issues

For the mtDNA final annotation we used primarily the GeSeq information. We found some disagreements regarding codon initiation and termination among the three softwares, which were corrected in the final consensus based on human (*Homo sapiens* NC_012920.1) and mouse (*Mus musculus* NC_005089.1) mitogenome alignments. In general, the annotation errors were the identification of the *ATP8* gene, which was identified as D-loop, and the *ND1* gene was reduced in size and was annotated in the wrong region. These disagreements highlight the importance of using more than one annotation software, to manually inspect and fix mistakes during the gene annotation procedure.

### Phylogenetic relationships within the genus *Trichechus*

One of the first studies that discussed trichechid diversification compared tooth and skeletal characteristics from extant *Trichechus* species with fossil taxa, and concluded that *T. manatus* and *T. senegalensis* have similar morphological characteristics—which could be the consequence of a close relationship, and that *T. inunguis* has more derived characteristics^3^. Other studies using molecular data found divergent results: a study using only the *cyt-b* sequence suggested a sister relationship between *T. manatus* and *T. senegalensis*^9^, while other studies using mitochondrial D-loop as a genetic marker found *T. senegalensis* as the sister group of *T. manatus* and *T. inunguis* with high support values^13,46^, similar to our data.

The divergence time estimates based on our mitogenomic data in the Family Trichechidae (approximately 7 Ma) are consistent with the fossil record for the genus in South America—register from Early Miocene and Late Miocene^3,47^. Notably, the history of *Trichechus* is difficult to explain looking only at living species, especially due the fact that its geographic distribution is very broad with species in the African continent, North America and South America. Also, we have few studies about its evolution using molecular data. Another genetic study^9^, that used D-loop as a marker, found a split between *T. inunguis* and *T. manatus* around 3.1–0.65 Ma, similar to our results.

### Evolutionary history of the genus *Trichechus*

The river courses on the South American continent underwent many modifications during the Neogene (23.03–2.5 Ma)^48–50^, which undoubtedly influenced the evolutionary history of the genus *Trichechus*. Based on our phylogenetic tree and divergence time estimates, we hypothesize a scenario for the evolutionary history of *Trichechus*, similar to a scenario suggested in the 1980s^3,10^.

**Figure 5:**
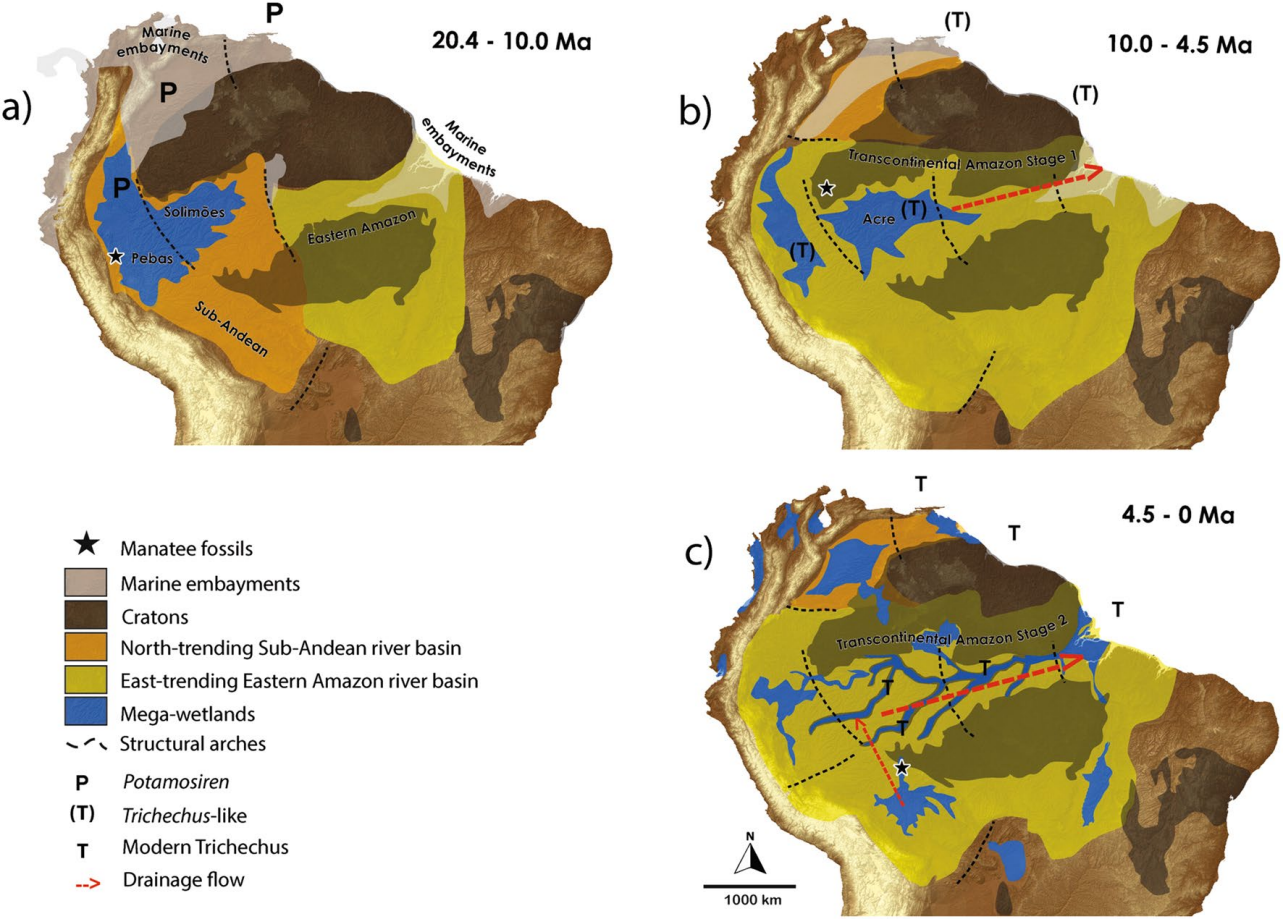
Schematic figure to show the evolution of the Amazon drainage pattern, based on Albert et al*.* (2018). (**a**) Early and Middle Miocene (20.4–9.0 Ma): *Potamosiren* (P) present in Magdalena basin based on Domning (1982), and fossil described during this period^66^; (**b**) Late Miocene (9.0–5.3 Ma): *Trichechus*-like sirenians (T) presumed to be present in the Amazon basin and South America Coast as suggested by Domning (1982), and fossil described by Beatty et al. (2012); (**c**) Middle Pliocene—Recent (c. 4.5–0 Ma): the possible occurrence of modern *Trichechus* species T, and a new species of *Trichechus* described recently by Perini et al*.* (2020).

During the formation of the Amazon Basin, the distribution and connections of wetlands had major influences on the evolution and diversity of many groups such as mammals, birds, reptiles and amphibians^51–55^. In the Early and Middle Miocene (20.4–10 Ma) there was an extensive wetland known as the Pebas Lake (Western Amazon) which was connected with the Caribbean Sea and associated with the higher sea-levels of the Mid-Miocene Climatic Optimum^48,56^ (Fig. 5a). It is possible that part of an ancestral lineage that later gave rise to *Trichechus* (*Potamosiren* REINHART, 1951, known from the Magdalena Basin of Colombia) inhabited both the Pebas Lake and the Caribbean Sea along the South American coast, which were connected^51^.

After this period, in the Late Miocene (9.0–5.3 Ma), the Pebas Lake changed due to tectonic movements (Andean Orogeny), received a huge amount of Andean-derived sediments, and is known as the Acre Lake, not connected to the Caribbean Sea^48,56^ (Fig. 5b). The manatees (*Trichechus*-like) that lived in the Acre Lake were at least semi-isolated in Western Amazonia from the other trichechids living along the South American Atlantic coast. Some authors^48^ indicate some overflow across the Purús Arch, providing a possible route for coastal manatees to swim upstream to the Acre Lake. These manatees likely became more adapted to the riverine environment, as evidenced by *Ribodon* AMEGHINO, 1883, considered the immediate ancestor of *Trichechus* and the first manatee to have had horizontally-replaced supernumerary teeth adapted to an abrasive diet. *Ribodon* is known only from the Rio Paraná basin in Argentina, and from North America, but it might have dispersed along the Atlantic coast and up the incipiently transcontinental Amazon River. Or, perhaps more likely, *Ribodon* could have evolved in the Acre Lake and dispersed downstream through the Rio Amazonas to the coast.

During the Plio-Pleistocene (5.3–0.012 Ma) the Andean-derived waters in the Acre Lake finally broke completely through the drainage divide into the Solimões and Amazonas sedimentary basins and discharged into the Atlantic Ocean, the modern situation^48,51^ (Fig. 5c). These nutrient-rich waters would have nourished abundant aquatic vegetation in these regions, including the abrasive true grasses (Gramineae or Poaceae) for which the supernumerary teeth of the manatees are adapted^6,10^. It is during this stage that we envision the evolution of the genus *Trichechus* and its diversification into *T. manatus* and *T. inunguis* that we know today, although these modern species maintain the ability to hybridize^46,48,51,57^. Other taxa now extinct may even have evolved in semi-isolated parts of the Amazon Basin; indeed, a new Late Pleistocene fossil, *Trichechus hesperamazonicus* PERINI et al., 2020, was recently discovered in a gold mine on the Madeira River (Brazil)^58^. It suggests that the phylogeny of manatees may prove to be more complex than is portrayed in this outline.

From among the *Trichechus* that at various times inhabited the Atlantic coast, it seems that the ancestors of modern African manatees reached West Africa by way of transoceanic currents from the Caribbean and South America as already suggested before^3,8,10^ and in accordance with our phylogenetic tree. From our analyses, we cannot specify when this dispersal might have happened, and as no trichechid fossils are known from Africa, this remains an open question. The discovery of more *Trichechu*s fossils in different locations, like the Caribbean region, the Amazon Basin, and Africa, seems indispensable to refine and eventually confirm this evolutionary scenario^10,58,59^.

Finally, it has been hypothesized that changes in climate and in the types of Caribbean seagrasses dominant during the Pliocene played a role in the extinction of the dugongs from the Caribbean area, which made possible the manatee expansion throughout that area; in fact, competition from manatees with more durable dentitions may have driven the last Caribbean dugongids to extinction^6,10^.

### Mitochondrial molecular adaptation in the genus *Trichechus*

Although most mitochondrial genes are conserved and evolved under purifying selection, several studies have reported that the action of positive selection in these genes is more common than previously thought^19,20,23^. Furthermore, mutations in mitochondrial genes can influence the production of reactive oxygen species in mammals^24^ and different implications for adaptive evolution of mitogenomes have been suggested in several studies. For example, selection in few branches (episodic selection) was related to niche change when a significantly higher ω ratio was found in mitochondrial genes of subterranean mammals^18,60^, freshwater dolphins^61^ and high-altitude alpacas^24^, and this might also be the case for *T. inunguis*.

Moreover, studies indicated that amino acid variations in mitogenome proteins may be related to functional implications such as adaptation to low-energy diet *versus* large body size and adaptation to extremely lowered O_2_ requirements in different mammal species^24^. Adaptation to temperature change was also related to mitogenome positive selection for hares^62^. In most studies, different subunits of the OXPHOS system I complex showed signs of positive selection, including *ND4*^18^. The *ND4* gene encodes one subunit of NADH dehydrogenase complex that is part of the oxidative phosphorylation machinery. This complex initiates the electrochemical proton gradient that leads to ATP synthesis. Hence, it has been suggested that mutations in these subunits may influence with the efficiency of the proton-pumping process^63–65^. We found one positively selected site located inside a critical protein region of the *ND4* OXPHOS subunit (site 181), suggesting that substitutions at this site may be adaptive, a finding that hints at its possible functional relevance. As a first step in the study of molecular evolutionary adaptations of these diving mammals, this result suggests the importance of developing a more in-depth and comparative study on the functionality of this subunit within the genus of *Trichechus*, which may confirm whether this variability represents an adaptive change related to *T. inunguis*. It is important to note that here we only investigated the mitochondrial genes related to the OXPHOS system; the nuclear genes that are part of this system remain to be tested.

## Conclusions

In summary, this first mitogenome phylogeny that includes all living *Trichechus* species provides a new framework for trichechid evolution, which was influenced by geological events in the formation of the Amazon Basin and by transoceanic currents. Probably the landscape change in the Amazon provided conditions for a population increase of *Trichechus* ancestors, which spread when the basin connected with the sea. We also showed evidence for positive selection acting in the *ND4 Trichechus inunguis* mtDNA gene. We suggest that this particular site might have functional implications related to metabolic efficiency in this species, but further experimental studies are needed to test this.

## Supplementary Information

Supplementary Information.
