# Supplementary material for: The evolutionary history of manatees told by their mitogenomes
Source: Sci Rep. 2021 Feb 11;11:3564. doi: 10.1038/s41598-021-82390-2 (PMC7878490; doi:10.1038/s41598-021-82390-2)
Supplement: Supplementary file 1 — Supplementary Information. [file 41598_2021_82390_MOESM1_ESM.docx]

**Full Title:** The evolutionary history of manatees told by their mitogenomes

**Short title: Origin and diversification of manatees**

Érica Martinha Silva de Souza ^1,*,^ ^¶^, Lucas Freitas ^1,^ ^¶^, Elisa Karen da Silva Ramos ^1,^ ^¶^, Giovanna Selleghin-Veiga ^1,&^, Michelle Carneiro Rachid Ribeiro ^1,&^, Felipe André Silva ^1^, Miriam Marmontel ^2^, Fabrício Rodrigues dos Santos ^3^, Anne Laudisoit ^4^, Erik Verheyen ^5,6^, Daryl P. Domning ^7,8^, Mariana Freitas Nery ^1,&,*^

**Supplementary material**


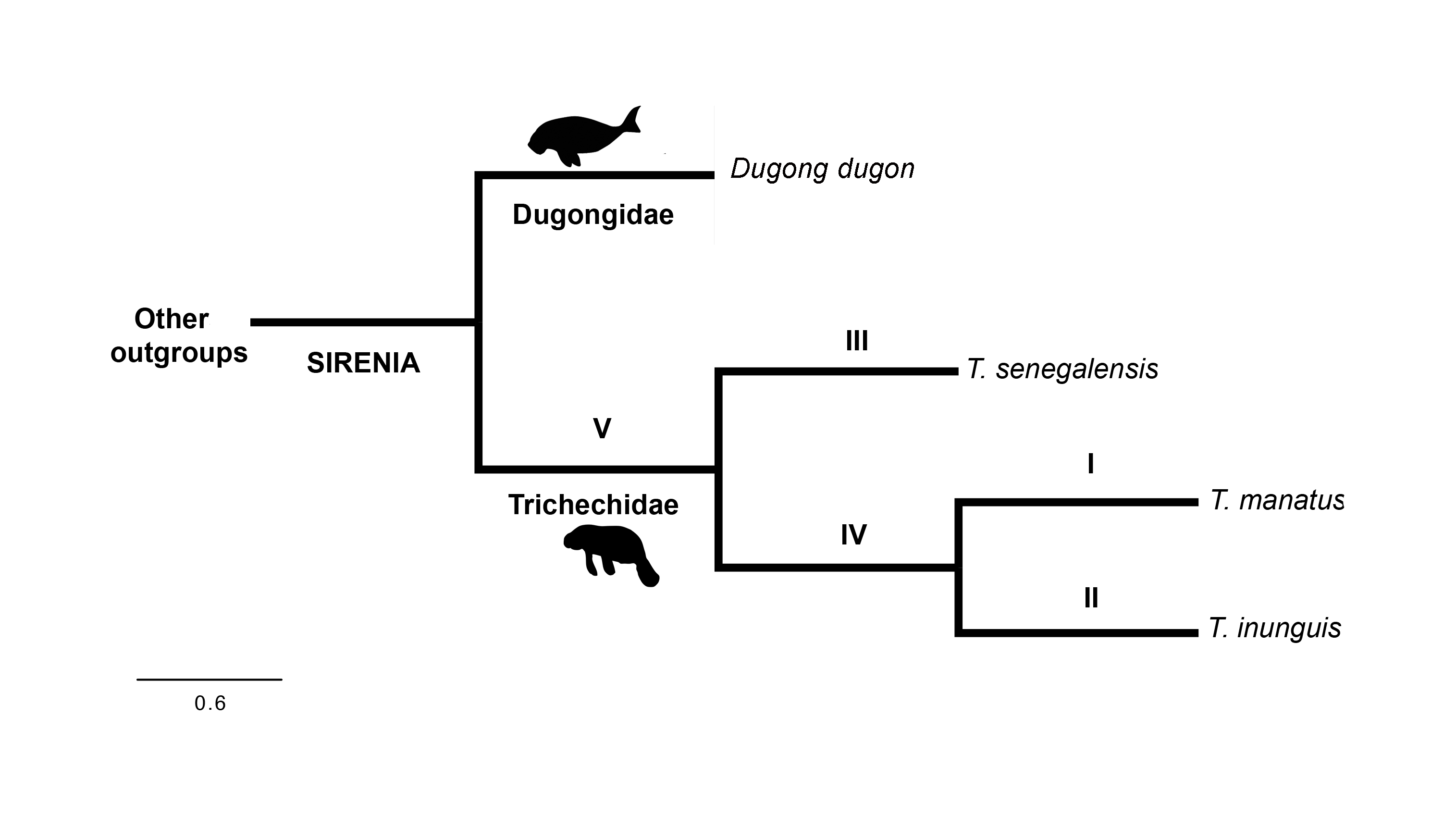


Figure 1. Cladogram showing the schemes of each branch tested in the analysis: I represents *T. manatus* selected; II *T. inunguis* selected; III *T. senegalensis* selected; IV only the Brazilian manatee branch selected; V represents all manatees selected.

Table 1. Comparative mitochondrial genome of the three manatee species, arrangement and annotation.

| Name | Type | Species | Start | End | Strand | Length (bp) | Start codon | Stop codon | Intron Codon |
| --- | --- | --- | --- | --- | --- | --- | --- | --- | --- |
| tRNA-Phe | tRNA | *T. manatus* | 1 | 70 | + | 71 |  |  | GAA |
|  |  | *T. inunguis* | 1 | 70 | + | 71 |  |  |  |
|  |  | *T. senegalensis* | 1 | 70 | + | 70 |  |  |  |
|  |  |  |  |  |  |  |  |  |  |
| 12S | rRNA | *T. manatus* | 71 | 1029 | + | 959 |  |  |  |
|  |  | *T. inunguis* | 71 | 1029 | + | 959 |  |  |  |
|  |  | *T. senegalensis* | 71 | 1029 | + | 959 |  |  |  |
|  |  |  |  |  |  |  |  |  |  |
| tRNA-Val | tRNA | *T. manatus* | 1030 | 1097 | + | 68 |  |  | TAC |
|  |  | *T. inunguis* | 1030 | 1097 | + | 68 |  |  |  |
|  |  | *T. senegalensis* | 1030 | 1097 | + | 68 |  |  |  |
|  |  |  |  |  |  |  |  |  |  |
| 16S | rRNA | *T. manatus* | 1098 | 2665 | + | 1568 |  |  |  |
|  |  | *T. inunguis* | 1098 | 2665 | + | 1568 |  |  |  |
|  |  | *T. senegalensis* | 1098 | 2667 | + | 1570 |  |  |  |
|  |  |  |  |  |  |  |  |  |  |
| tRNA-Leu | tRNA | *T. manatus* | 2666 | 2740 | + | 75 |  |  | TAA |
|  |  | *T. inunguis* | 2666 | 2740 | + | 75 |  |  |  |
|  |  | *T. senegalensis* | 2668 | 2742 | + | 75 |  |  |  |
|  |  |  |  |  |  |  |  |  |  |
| ND1 | coding | *T. manatus* | 2741 | 3697 | + | 957 | GTG | TAA |  |
|  |  | *T. inunguis* | 2741 | 3697 | + | 967 | GTG | TAA |  |
|  |  | *T. senegalensis* | 2743 | 3697 | + | 955 | GTG | CAT |  |
|  |  |  |  |  |  |  |  |  |  |
| tRNA-Ile | tRNA | *T. manatus* | 3698 | 3766 | + | 69 |  |  | GAT |
|  |  | *T. inunguis* | 3698 | 3766 | + | 69 |  |  |  |
|  |  | *T. senegalensis* | 3698 | 3768 | + | 71 |  |  |  |
|  |  |  |  |  |  |  |  |  |  |
| tRNA-Gln | tRNA | *T. manatus* | 3764 | 3835 | - | 72 |  |  | TTG |
|  |  | *T. inunguis* | 3764 | 3835 | - | 72 |  |  |  |
|  |  | *T. senegalensis* | 3766 | 3837 | - | 72 |  |  |  |
|  |  |  |  |  |  |  |  |  |  |
| tRNA-Met | tRNA | *T. manatus* | 3840 | 3908 | + | 69 |  |  | CAT |
|  |  | *T. inunguis* | 3840 | 3908 | + | 69 |  |  |  |
|  |  | *T. senegalensis* | 3842 | 3910 | + | 69 |  |  |  |
|  |  |  |  |  |  |  |  |  |  |
| ND2 | coding | *T. manatus* | 3909 | 4950 | + | 1042 | ATC | AAT |  |
|  |  | *T. inunguis* | 3909 | 4950 | + | 1042 | ATC | AAT |  |
|  |  | *T. senegalensis* | 3911 | 4952 | + | 1042 | ATC | AAT |  |
|  |  |  |  |  |  |  |  |  |  |
| tRNA-Trp | tRNA | *T. manatus* | 4951 | 5019 | + | 69 |  |  | TCA |
|  |  | *T. inunguis* | 4951 | 5019 | + | 69 |  |  |  |
|  |  | *T. senegalensis* | 4953 | 5021 | + | 69 |  |  |  |
|  |  |  |  |  |  |  |  |  |  |
| tRNA-Ala | tRNA | *T. manatus* | 5023 | 5091 | - | 69 |  |  | TGC |
|  |  | *T. inunguis* | 5023 | 5091 | - | 69 |  |  |  |
|  |  | *T. senegalensis* | 5025 | 5093 | - | 69 |  |  |  |
|  |  |  |  |  |  |  |  |  |  |
| tRNA-Asn | tRNA | *T. manatus* | 5092 | 5164 | - | 73 |  |  | GTT |
|  |  | *T. inunguis* | 5092 | 5164 | - | 73 |  |  |  |
|  |  | *T. senegalensis* | 5094 | 5166 | - | 73 |  |  |  |
|  |  |  |  |  |  |  |  |  |  |
| tRNA-Cys | tRNA | *T. manatus* | 5200 | 5265 | - | 66 |  |  | GCA |
|  |  | *T. inunguis* | 5200 | 5265 | - | 66 |  |  |  |
|  |  | *T. senegalensis* | 5202 | 5267 | - | 66 |  |  |  |
|  |  |  |  |  |  |  |  |  |  |
| tRNA-Tyr | tRNA | *T. manatus* | 5266 | 5332 | - | 67 |  |  | GTA |
|  |  | *T. inunguis* | 5265 | 5333 | - | 69 |  |  |  |
|  |  | *T. senegalensis* | 5268 | 5334 | - | 67 |  |  |  |
|  |  |  |  |  |  |  |  |  |  |
| COX1 | coding | *T. manatus* | 5334 | 6875 | + | 1542 | ATG | TAG |  |
|  |  | *T. inunguis* | 5334 | 6875 | + | 1542 | ATG | TAG |  |
|  |  | *T. senegalensis* | 5336 | 6877 | + | 1542 | ATG | TAG |  |
|  |  |  |  |  |  |  |  |  |  |
| tRNA-Ser | tRNA | *T. manatus* | 6878 | 6946 | - | 69 |  |  | TGA |
|  |  | *T. inunguis* | 6876 | 6944 | - | 69 |  |  |  |
|  |  | *T. senegalensis* | 6880 | 6948 | - | 69 |  |  |  |
|  |  |  |  |  |  |  |  |  |  |
| tRNA-Asp | tRNA | *T. manatus* | 6954 | 7022 | + | 69 |  |  | GTC |
|  |  | *T. inunguis* | 6954 | 7022 | + | 69 |  |  |  |
|  |  | *T. senegalensis* | 6956 | 7024 | + | 69 |  |  |  |
|  |  |  |  |  |  |  |  |  |  |
| COX2 | coding | *T. manatus* | 7023 | 7706 | + | 684 | ATG | TAA |  |
|  |  | *T. inunguis* | 7023 | 7706 | + | 684 | ATG | TAA |  |
|  |  | *T. senegalensis* | 7025 | 7708 | + | 684 | ATG | TAA |  |
|  |  |  |  |  |  |  |  |  |  |
| tRNA-Lys | tRNA | *T. manatus* | 7709 | 7775 | + | 67 |  |  | TTT |
|  |  | *T. inunguis* | 7707 | 7775 | + | 69 |  |  |  |
|  |  | *T. senegalensis* | 7711 | 7777 | + | 67 |  |  |  |
|  |  |  |  |  |  |  |  |  |  |
| ATPase 8 | coding | *T. manatus* | 7779 | 7984 | - | 206 | CCT | TGG |  |
|  |  | *T. inunguis* | 7781 | 7776 | - | 206 | ATG | TAA |  |
|  |  | *T. senegalensis* | 7778 | 7981 | - | 204 | ATG | TAA |  |
|  |  |  |  |  |  |  |  |  |  |
| ATPase 6 | coding | *T. manatus* | 7942 | 8620 | + | 679 | AAC | TAA |  |
|  |  | *T. inunguis* | 7939 | 8619 | + | 681 | ATG | TAA |  |
|  |  | *T. senegalensis* | 7939 | 8617 | + | 679 | ATG | CCT |  |
|  |  |  |  |  |  |  |  |  |  |
| COX3 | coding | *T. manatus* | 8619 | 9402 | + | 784 | ATG | CCT |  |
|  |  | *T. inunguis* | 8619 | 9402 | + | 784 | ATG | CCT |  |
|  |  | *T. senegalensis* | 8619 | 9402 | + | 784 | ATG | CCT |  |
|  |  |  |  |  |  |  |  |  |  |
| tRNA-Gly | tRNA | *T. manatus* | 9403 | 9471 | + | 69 |  |  | TCC |
|  |  | *T. inunguis* | 9402 | 9471 | + | 70 |  |  |  |
|  |  | *T. senegalensis* | 9403 | 9471 | + | 69 |  |  |  |
|  |  |  |  |  |  |  |  |  |  |
| ND3 | coding | *T. manatus* | 9472 | 9818 | + | 347 | ATT | ATA |  |
|  |  | *T. inunguis* | 9472 | 9818 | + | 347 | ATT | ATA |  |
|  |  | *T. senegalensis* | 9472 | 9818 | + | 347 | ATT | ATA |  |
|  |  |  |  |  |  |  |  |  |  |
| tRNA-Arg | tRNA | *T. manatus* | 9819 | 9885 | + | 67 |  |  | TCG |
|  |  | *T. inunguis* | 9819 | 9885 | + | 67 |  |  |  |
|  |  | *T. senegalensis* | 9819 | 9885 | + | 67 |  |  |  |
|  |  |  |  |  |  |  |  |  |  |
| ND4L | coding | *T. manatus* | 9888 | 10184 | + | 297 | ATG | TAA |  |
|  |  | *T. inunguis* | 9888 | 10184 | + | 297 | ATG | TAA |  |
|  |  | *T. senegalensis* | 9888 | 10184 | + | 297 | ATG | TAA |  |
|  |  |  |  |  |  |  |  |  |  |
| ND4 | coding | *T. manatus* | 10178 | 11555 | + | 1,378 | ATG | TCT |  |
|  |  | *T. inunguis* | 10178 | 11555 | + | 1,378 | ATG | TCT |  |
|  |  | *T. senegalensis* | 10178 | 11555 | + | 1378 | ATG | TCT |  |
|  |  |  |  |  |  |  |  |  |  |
| tRNA-His | tRNA | *T. manatus* | 11556 | 11624 | + | 69 |  |  | GTG |
|  |  | *T. inunguis* | 11556 | 11624 | + | 69 |  |  |  |
|  |  | *T. senegalensis* | 11556 | 11624 | + |  |  |  |  |
|  |  |  |  |  |  |  |  |  |  |
| tRNA-Ser | tRNA | *T. manatus* | 11625 | 11683 | + | 59 |  |  | GCT |
|  |  | *T. inunguis* | 11625 | 11683 | + | 59 |  |  |  |
|  |  | *T. senegalensis* | 11625 | 11683 | + | 59 |  |  |  |
|  |  |  |  |  |  |  |  |  |  |
| tRNA-Leu | tRNA | *T. manatus* | 11684 | 11753 | + | 70 |  |  | TAG |
|  |  | *T. inunguis* | 11684 | 11753 | + | 70 |  |  |  |
|  |  | *T. senegalensis* | 11684 | 11753 | + | 70 |  |  |  |
|  |  |  |  |  |  |  |  |  |  |
| ND5 | coding | *T. manatus* | 11754 | 13565 | + | 1812 | ATT | TAA |  |
|  |  | *T. inunguis* | 11754 | 13565 | + | 1812 | ATT | TAA |  |
|  |  | *T. senegalensis* | 11754 | 13565 | + | 1812 | ATT | TAA |  |
|  |  |  |  |  |  |  |  |  |  |
| ND6 | coding | *T. manatus* | 13562 | 14086 | - | 525 | CTA | CAT |  |
|  |  | *T. inunguis* | 13562 | 14079 | - | 528 | CTA | CAT |  |
|  |  | *T. senegalensis* | 13562 | 14089 | - | 528 | TAC | ATC |  |
|  |  |  |  |  |  |  |  |  |  |
| tRNA-Glu | tRNA | *T. manatus* | 14090 | 14158 | - | 69 |  |  | TTC |
|  |  | *T. inunguis* | 14080 | 14148 | - | 69 |  |  |  |
|  |  | *T. senegalensis* | 14090 | 14158 | - | 69 |  |  |  |
|  |  |  |  |  |  |  |  |  |  |
| CYTB | coding | *T. manatus* | 14164 | 15302 | + | 1139 | ATG | AGG |  |
|  |  | *T. inunguis* | 14164 | 15302 | + | 1139 | ATG | AGG |  |
|  |  | *T. senegalensis* | 14164 | 15302 | + | 1139 | ATG | AAG |  |
|  |  |  |  |  |  |  |  |  |  |
| tRNA-Thr | tRNA | *T. manatus* | 15303 | 15369 | + | 67 |  |  | TGT |
|  |  | *T. inunguis* | 15303 | 15369 | + | 67 |  |  |  |
|  |  | *T. senegalensis* | 15303 | 15369 | + | 67 |  |  |  |
|  |  |  |  |  |  |  |  |  |  |
| tRNA-Pro | tRNA | *T. manatus* | 15371 | 15437 | - | 67 |  |  | TGG |
|  |  | *T. inunguis* | 15371 | 15437 | - | 67 |  |  |  |
|  |  | *T. senegalensis* | 15371 | 15437 | - | 67 |  |  |  |
|  |  |  |  |  |  |  |  |  |  |
| D-Loop |  | *T. manatus* | 15438 | 16930 |  | 1493 |  |  |  |
|  |  | *T. inunguis* | 15438 | 16930 |  | 1493 |  |  |  |
|  |  | *T. senegalensis* | 15438 | 16882 |  | 1445 |  |  |  |


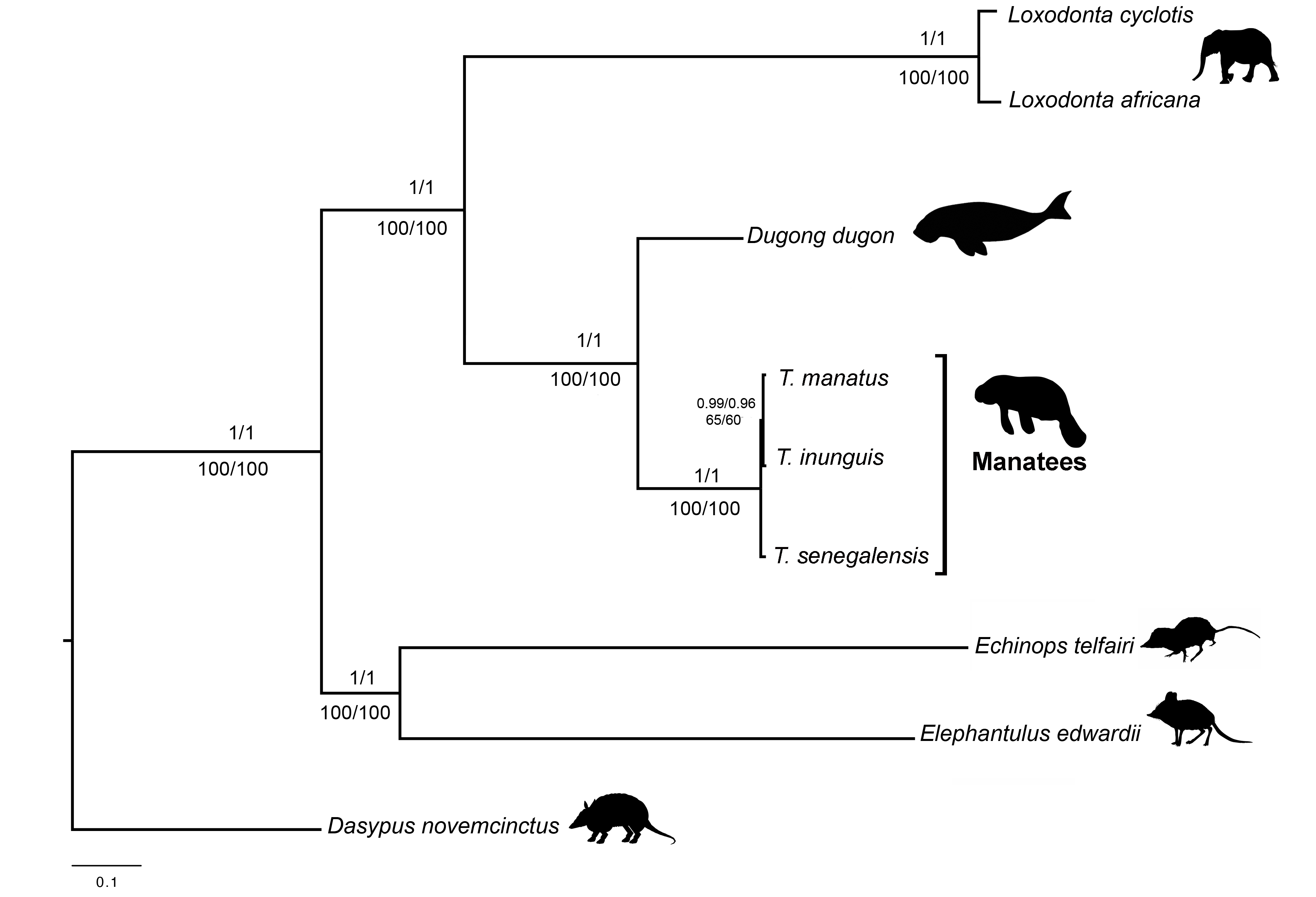


Figure 2. Topology resulting from Bayesian and maximum likelihood analysis. The numbers above the branches represent the posterior probability (pp), and numbers below the branches represent the bootstrap. The values on the left side represent results from all mtDNA analyses, and on the right side the analysis results using the cds.
